# Begonia—A Two-Photon Imaging Analysis Pipeline for Astrocytic Ca^2+^ Signals

**DOI:** 10.3389/fncel.2021.681066

**Published:** 2021-05-20

**Authors:** Daniel M. Bjørnstad, Knut S. Åbjørsbråten, Eivind Hennestad, Céline Cunen, Gudmund Horn Hermansen, Laura Bojarskaite, Klas H. Pettersen, Koen Vervaeke, Rune Enger

**Affiliations:** ^1^GliaLab at the Letten Centre, Division of Anatomy, Department of Molecular Medicine, Institute of Basic Medical Sciences, University of Oslo, Oslo, Norway; ^2^Lab for Neural Computation, Division of Physiology, Department of Molecular Medicine, Institute of Basic Medical Sciences, University of Oslo, Oslo, Norway; ^3^Statistics and Data Science Group, Department of Mathematics, Faculty of Mathematics and Natural Sciences, University of Oslo, Oslo, Norway; ^4^Department of Neurology, Oslo University Hospital, Rikshospitalet, Oslo, Norway; ^5^NORA—Norwegian Artificial Intelligence Research Consortium, Faculty of Mathematics and Natural Sciences, University of Oslo, Oslo, Norway

**Keywords:** two-photon (2P), image analysis, calcium imaging, ROA analysis, astrocyte

## Abstract

Imaging the intact brain of awake behaving mice without the dampening effects of anesthesia, has revealed an exceedingly rich repertoire of astrocytic Ca^2+^ signals. Analyzing and interpreting such complex signals pose many challenges. Traditional analyses of fluorescent changes typically rely on manually outlined static region-of-interests, but such analyses fail to capture the intricate spatiotemporal patterns of astrocytic Ca^2+^ dynamics. Moreover, all astrocytic Ca^2+^ imaging data obtained from awake behaving mice need to be interpreted in light of the complex behavioral patterns of the animal. Hence processing multimodal data, including animal behavior metrics, stimulation timings, and electrophysiological signals is needed to interpret astrocytic Ca^2+^ signals. Managing and incorporating these data types into a coherent analysis pipeline is challenging and time-consuming, especially if research protocols change or new data types are added. Here, we introduce Begonia, a MATLAB-based data management and analysis toolbox tailored for the analyses of astrocytic Ca^2+^ signals in conjunction with behavioral data. The analysis suite includes an automatic, event-based algorithm with few input parameters that can capture a high level of spatiotemporal complexity of astrocytic Ca^2+^ signals. The toolbox enables the experimentalist to quantify astrocytic Ca^2+^ signals in a precise and unbiased way and combine them with other types of time series data.

## Introduction

Astrocytic Ca^2+^ signals have been shown to play important roles in a wide range of physiological and pathophysiological brain processes (Cornell-Bell et al., 1990; Rusakov et al., 2011; Verkhratsky and Parpura, 2013; Bazargani and Attwell, 2016). Until recently, studies on astrocytic Ca^2+^ signals were confined to *in vitro* experiments and *in vivo* experiments in anesthetized mice. With the development of genetically encoded Ca^2+^ sensors and improvements in optical imaging, *in vivo* imaging without the dampening effects of anesthesia (Thrane et al., 2012) in behaving animals has become possible (Srinivasan et al., 2015; Enger et al., 2017; Stobart et al., 2018). This, in turn, has revealed an exceedingly rich palette of astrocytic Ca^2+^ signals coupled to behavior, ranging from small, short-lived events in subcellular compartments to long-lasting, global activations affecting large swaths of the cortical mantle (Srinivasan et al., 2015; Stobart et al., 2018; Bojarskaite et al., 2020).

High frame rates (e.g., ~30 Hz) are needed to appropriately capture short-lasting Ca^2+^ signals and enable efficient movement correction of two-photon imaging data from awake mice (Pnevmatikakis and Giovannucci, 2017; Stobart et al., 2018). Moreover, to reliably quantify the relatively sparse astrocytic Ca^2+^ signals in behavioral quiescence, long acquisition times are also warranted. For these reasons, two-photon microscopy data for a given project could amount to tens of terabytes of data, and an analysis pipeline for such datasets needs to be optimized for speed and performance to avoid unacceptably long processing times.

Traditional analyses of astrocytic Ca^2+^ signals typically comprise manual or semi-automatic segmentation of regions-of-interest (ROIs) overlying astrocytic somata and processes (Eilert-Olsen et al., 2019; Semyanov et al., 2020). Even though such analyses are appropriate and to some level sufficient to describe the relatively sparse astrocytic Ca^2+^ activity in brain slices and the anesthetized brain, they are not adequate for capturing the true complexity of astrocytic Ca^2+^ signals in the unanesthetized brain (Wang et al., 2019; Bojarskaite et al., 2020; Semyanov et al., 2020): first, ROI analyses do not capture the dynamic spatial extent of these signals, as astrocytic Ca^2+^ signals can emerge from multiple point sources, merge, and spread throughout the gap junction coupled astrocyte syncytium (Semyanov, 2019). Second, as fluorescence from an ROI is typically measured as the mean gray value of the pixels within that ROI per time unit, events affecting a small proportion of the segmented area will typically not reach the threshold for event detection. Third, a static ROI analysis fails to capture separate concurrent events occurring within a single defined area (Wang et al., 2019; Bojarskaite et al., 2020). Lately, new algorithms have been proposed to appropriately address the dynamic nature of astrocytic Ca^2+^ signaling (Srinivasan et al., 2015; Barrett et al., 2018; Wang et al., 2019). One of these, the *Astrocyte Quantitative Analysis* (AQuA) algorithm, employs an event-based approach to detect astrocytic Ca^2+^ signals (Wang et al., 2019). AQuA has in our view prominently advanced the field of astroglial Ca^2+^ signal analyses, especially by its efforts to describe how astroglial Ca^2+^ signals dynamically change in time and space. Even so, AQuA is dependent on a wide range of tuning parameters, and the analysis pipeline is not optimized for high frame rate data (Bojarskaite et al., 2020).

Another challenge with analyzing astrocytic Ca^2+^ signaling data from unanesthetized awake–behaving mice is to properly align and interpret these in the context of rich animal behavior. Various time series data are acquired for studies in behaving animals that need to be integrated with astrocytic signaling. For example, locomotion and whisking activity are typically recorded. To properly collate and align such multimodal data is challenging as they are typically acquired by multiple recording devices with different sampling frequencies and data formats.

Here, we present a MATLAB toolbox tailored to analyze astrocytic Ca^2+^ signals from behaving animals in a timely manner. The toolbox comprises a data management pipeline from raw data to derived data in tables, is optimized for large datasets, and contains the following functions: (i) implementation of previously published image alignment algorithms (Pnevmatikakis and Giovannucci, 2017); (ii) an automatic Ca^2+^ signal analysis pipeline, the region-of-activity (ROA) method, that may be used without setting manual tuning parameters; (iii) an ROI segmentation graphical user interface that can combine hand-drawn ROIs with automatically detected Ca^2+^ signals; (iv) easy integration with other time series data such as electrophysiological recordings or movement data; and (v) an output module that can export data as tables for statistical analyses, or as plots and figures. The toolbox is programmed in MATLAB with modularity and flexibility in mind, enabling quick creation of graphical user interfaces and workflows when new analyses need to be established.

## Materials and Methods

The Begonia toolbox comprises multiple graphical and programmatic tools placed on top of a framework for the storage of metadata and derived data from image recordings, and a set of abstract base classes that offer a common application programming interface (API) for accessing image data in a source agnostic manner.

### Metadata Storage With Data Locations

We define metadata as all data connected to the recording and analysis of a two-photon microscopy experiment except the actual imaging data. We developed Begonia around a metadata storage strategy we call *data locations* which is facilitated by the DataLocation class. Data locations offer a way for the software provided by Begonia to easily store and retrieve metadata as processing and manual steps take place on imaging data.

In simple terms, data locations are paths in the file directory that through the DataLocation class allow metadata variables to be associated with these paths. The system does not rely on a centralized database of any kind and does not enforce a strict schema of names and entities on the data being saved. Data locations by default store the metadata entries at the path they point to. A problem with using filesystem paths as identifiers is to keep track of the data if files and folders are moved. For this reason, data locations save a universal unique identifier (UUID) on first use so that data that are moved can be re-identified.

The data location system supports several ways of storing and retrieving metadata through the use of keywords, with abstract access to these mechanisms through a generic API on the DataLocation objects. We provide two such methods, or *engines*: the on-path engine, and the off-path engine. The on-path engine is the default, and stores metadata in a .mat file adjacent to the imaging data. The off-path engine stores all the metadata in a separate directory chosen by the user. Using the on-path engine, data locations require no setup to attach additional data to the imaging data.

### Data Import, Management, and On-Demand Reading

We offer an API for importing imaging data and metadata acquired by different microscopes into a standardized format. This is implemented through the TSeries class, which provides a gateway to use the functionality of Begonia, regardless of how the data are recorded. Begonia contains a +scantype namespace that contains classes that inherit from the TSeries class that can load multi-page TIFF files [an adaptation of the TIFFStack (Muir and Kampa, 2014) library for faster loading and alternative matrix indexing], PrairieView (Bruker) time series, and TIFFs created in ScanImage (Vidrio Technologies). The imaging classes also inherit the DataLocation class, allowing new metadata to be associated directly with a particular recording and granting access to the data management system.

We have frequently found that two-photon microscopy data from a single trial exceed the random-access memory size (RAM) on the analyst’s computer. Moreover, we often want to review specific moments without waiting to load the whole recording. To allow faster and improved loading, we convert large data to the Hierarchical Data Format 5 (HDF5) whenever possible. HDF5 allows data to be retrieved on-demand, utilizing only the memory needed for the operation in question and retrieving only a subset of the recording of interest.

**Figure 1:**
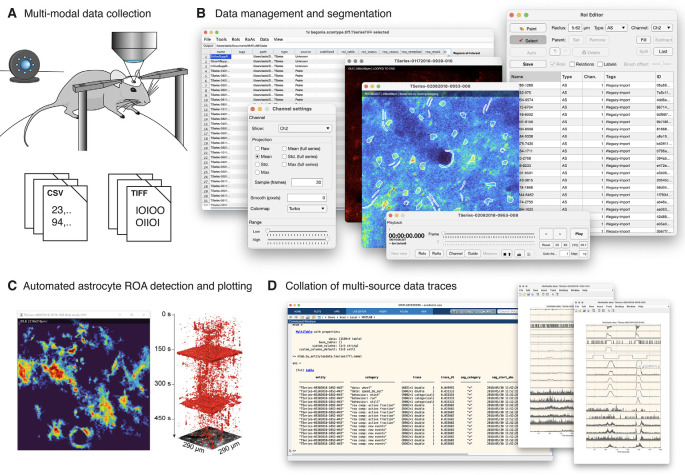
Begonia workflow. **(A)** Acquisition of multi-modal data. Movement from a running wheel and surveillance video from an infrared-sensitive camera is typically recorded alongside two-photon imaging data. **(B)** Begonia provides an editor for project-specific manual tasks, data and metadata management, and manual ROI segmentation of the imaging data. **(C)** The region-of-activity (ROA) algorithm provides an automated or semi-automatic detection of astrocytic Ca^2+^ signals in the field-of-view or inside manually segmented ROIs or groups of manually segmented ROIs. **(D)** MultiTable provides a way to collate, synchronize and segment time series traces from ROIs, ROA, and custom sources on demand.

Analysts can use imported recordings directly in MATLAB. However, Begonia offers a data management tool, that lists recordings and displays associated metadata through the data location system (Figure 1B). The tool offers multiple features such as filtering, processing, and plotting through simple drop-down menus and buttons. The data manager is built on a base class called Editor that allows programmers to quickly develop one-shot graphical user interfaces (GUIs) with customized visualization of data and metadata as well as buttons for project-specific processing functions. We find that GUIs and the DataLocation system enable an efficient and flexible analysis pipeline that provides non-expert programmers with easy access to a complex processing pipeline. The GUIs are particularly useful for performing manual steps of the analyses, such as marking anatomical features and reviewing the data quality of individual recordings. Manually generated metadata can be used by processing functions that subsequently can be executed directly or added to a processing queue from the data manager.

### Collation and Segmentation of Time Series Data

Once data is processed, it is often necessary to combine them with other data types, like for instance recordings of locomotion and whisking. Such additional data are typically acquired on a different computer than the imaging data and are often of different acquisition rates, starting times, and data formats than the microscopy data. It is useful to plot or analyze all these multimodal data in a collated fashion. To facilitate this, Begonia implements a class called MultiTable and an associated API that can collect data from multiple sources and group them by a custom entity such as an experiment or trial identifier. In this way, all data from a single experiment are connected using a common entity.

MultiTable is not a table of data, but a list of sources that provides data in a uniform tabular format on demand. It can additionally slice and resample the data based on user-given criteria. With this approach, the origin of the data can be hidden from the user and thus simplify the analysis. We provide sources for data locations out of the box, which simplifies adding data from ROI and ROA analyses. The sources must provide time information in addition to the data vectors themselves. With this requirement, analysts can resample and time-align MultiTable outputs. Additionally, all data provided to MultiTable can be added with time correction so that differences in starting time on different hardware can be compensated. The system provides a way to include any custom time series data alongside the data types provided by Begonia.

We frequently need to extract time series data from various data sources around a time point or in a specified time interval. The MultiTable API allows such segmentation using MATLAB categorical arrays. For example, it may be desirable to extract fluorescence values and whisking activity around the transition from quiet wakefulness to running. In this case, the categorical array in the MultiTable (Figure 1D) could contain the value “quiet wakefulness” in the time points where the mouse is still, and “running” when the mouse moves. MultiTable can then be asked to identify these transitions based on the categorical arrays and retrieve data from an interval relative to such a time point.

### ROI Manager

In addition to metadata and data management features, Begonia provides a highly modular ROI management and time series viewer called ROI Manager. The software is designed to be extended for any type of markup a project might need, and to be a generic viewer for data loaded through Begonia.

Architecturally, ROI Manager uses a central concept of *Views* for windows that display data, and *Tools* for windows that provide tools to edit the data. It is designed with a hybrid object and data-oriented design. Each piece of data loaded—primarily two-photon microscopy time series data—can have multiple views of the same imaging data simultaneously. In this way, imaging data from multiple channels may be viewed simultaneously in different windows. *Views* provide a data-oriented approach to communication between the modules of the ROI Manager (tools, rendering layers, and, interaction modes) by offering a set of *key-value pairs* (KVPs) for each *View* that can be read and written by any module and periodically checked. The KVPs have non-strict semantics, meaning modules can read and write any data they want to the *Views*. E.g., an ROI editing tool can write a table of ROIs to the key “roi_table,” and a tool for changing the colormap displayed can write these settings to a key called “channel_colormap,” while both can write to a key “channel” to set what channel in the multi-channel data is currently displayed. The modules handling the rendering of the data in the figure window similarly check if keys they are associated with are updated and then redraw the view of the data as needed.

Tools and various modules can be set up to load in any combination the user wants, allowing the ROI Manager to take roles beyond managing ROIs. For instance, a project measuring vascular diameters and flow of red blood cells might warrant a Tool window for marking the vessel, in combination with visible ROIs. In its simplest form, ROI Manager is just an image sequence viewer with no Tools, and Begonia offers this as well to allow viewing any 3D MATLAB arrays.

Begonia offers ROI signal extraction from ROIs in time series data through batch operations available in Data Manager. In addition, the ROIs from the ROI Manager may be used to filter the output of the ROA analysis to assign ROA activity to cells or subcellular structures.

### ROA Algorithm

**Figure 2:**
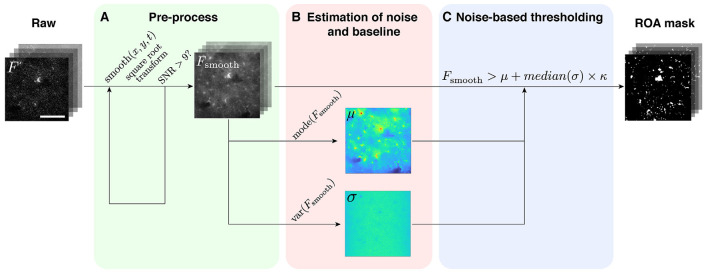
The ROA algorithm. **(A)** The raw data (*F*) are spatiotemporally smoothed and transformed (*F*_smooth_) until an acceptable SNR is attained. **(B)** In each pixel, the baseline value (μ) and the standard deviation (σ) of the root gray values are then estimated from *F*_smooth_ (temporal smoothing applied by merging frames). **(C)** *F*_smooth_ is then binarized by applying a threshold defined as the baseline value (μ) plus *κ* times the standard deviation of the noise (σ; temporal smoothing applied with a moving average).

The ROA algorithm is a method that detects fluorescence signal events in a pixel-by-pixel fashion in two-photon microscopy time series data (Figure 2). The raw fluorescence time series (*F*) of each pixel is transformed into a binary time series where the ones indicate events. These events correspond to fluorescence values which exceed a certain pixel-specific threshold *τ_i_*. The threshold is a function of the baseline gray values and standard deviation of the noise. The algorithm is tailor-made for noisy, high frame rate (~30 Hz) recordings.

### Finding Activity

We denote the raw fluorescence time series as *F*_i_(*t*), i.e., the gray value of pixel *i* at time *t*. Let $F_{i}^{'}(t)$ denote a moving average smoothed version of *F*_i_(*t*); (see below for details on smoothing), and consider $F_{i}^{*}(t)=\sqrt{F_{t}^{'}}(t)$ the square root transformed version of $F_{i}^{'}(t)$. We determine events in our time series data, by binarizing $F_{i}^{*}(t)$ per pixel using a threshold ( *τ_i_*) defined as:

$$\tau_{i}={\hat{\mu }}_{i}+\kappa \cdot \hat{\sigma },$$

where *κ* is a user-defined number (default *κ* = 4) which determines the height of the threshold, ${\hat{\mu }}_{i}$ is the estimated baseline value for each pixel, and $\hat{\sigma }$ is the estimated standard deviation of the noise as defined in the following sections.

### Modeling Fluorescence Time Series

To accurately estimate the level of noise, one needs to model the underlying distribution of the fluorescence data. When the number of photons recorded per pixel is sufficiently high, fluorescence data from two-photon Ca^2+^ imaging may be considered approximately normally distributed with a particular relationship between the expected value and the variance; *Var_i_*(*t*) = *aE_i_*(*t*) + *b*, with unknown parameters *a* and *b*, due to the effect of the amplification of the signal by the photomultiplier tubes (Danielyan et al., 2014). Here *E_i_*(*t*) denotes the expected fluorescence value at time *t* and in a particular pixel *i* and *Var_i_*(*t*) the variance of the fluorescence. Our raw, high frame rate data (*F_i_*(*t*)) have a highly non-normal distribution, with a high probability of observing values equal to zero and a heavy right tail. After smoothing (mainly in time), we observe that the distribution comes closer to normality. This is natural, since smoothing has a similar effect on the distribution of the pixel gray values as increasing the pixel integration time. At this level of the analysis, we perform spatiotemporal smoothing by averaging a specific number of frames and neighboring pixels and we denote this time series by *Z_i_*(*t*). The particular dependency between the expected value and variance in the model of Danielyan et al. (2014) may be essentially eliminated using the following transformation $X_{i}(t)=\sqrt{Z_{i}(t)}$. The transformed variable *X_i_*(*t*) will have a time-varying expected value $\mu_{i}(t)\approx \sqrt{E(Z_{i}(t))}$ and an (essentially) time-independent variance $\sigma_{i}^{2}$. In the following, *E* denotes the expected value and *σ_i_* represents the standard deviation of the noise. The transformation above is occasionally referred to as a variance stabilization in the literature (Bartlett, 1947; Wang et al., 2019). Note that the variance after transformation will, in fact, be approximately equal to the following expression

$$Var(X_{i}(t))\approx a/4+b/[4E(Z_{i}(t))].$$

This expression can be derived using the delta method, see for instance Casella and Berger (2002). Clearly, the dependency between the variance and the expected value will be negligible if the expected value, *E*(*Z_i_*(*t*)), is sufficiently large, and after the square root transformation, we observe that there is virtually no dependency between μ_i_(*t*) and *Var*(*X_i_*(*t*)), which suggests that *b* is an order of magnitude smaller than *E*(*Z_i_*(*t*)). We therefore ignore *b* in the following.

### Estimating Pixel-Specific Parameters

Estimating the time-varying expectation μ_i_(*t*) requires additional assumptions and can be challenging. Furthermore we are primarily interested in estimating the baseline of *X_i_*(*t*), which we refer to as μ_i_. We estimate this quantity by finding the mode of the observations in pixel *i*,

$${\hat{\mu }}_{i}=Mode(X_{i}).$$

Intuitively, since the fluorescence signal in each pixel primarily takes values close to the baseline for most of the recording, it is natural to estimate the baseline by the most frequent value in the time series for pixel *i*. In our investigations, this estimate appears to work adequately.

Since we do not estimate the time-varying expected value μ_i_(*t*), we cannot use the conventional sample variance formula. Instead, we use the following estimator for the variance of the noise,

$${\hat{\sigma }}_{i}^{2}=\frac{1}{c}Median\;({\left[X_{i}(t+1)-X_{i}(t)\right]}^{2}),$$

for all pairs of successive observations. The number *c* is a constant which ensures that this is a consistent estimator for $\sigma_{i}^{2}$ under the assumption of normality. The value of *c* may be found by simulations or calculated theoretically, by

$$c=2m_{1}\approx 0.9099,$$

where *m*_1_ is the median of the chi-squared distribution with 1 degree of freedom. This formula for c is an approximation that holds when the number of time points is sufficiently large. This estimator was employed by Wang et al. (2019). Its theoretical properties have not been derived as far as we know, but its form and motivation is close to the mean squared successive difference (MSSD) estimator suggested by Neumann et al. (1941) and with a relatively widespread use (Ebner-Priemer et al., 2007, 2009; Garrett et al., 2013; Nomi et al., 2017).

### Smoothing

The number of events detected with the ROA algorithm is dependent on the imaging quality, which can be assessed by the signal-to-noise ratio (SNR), which we define as

$$SNR=\hat{\mu }/\hat{\sigma },$$

where $\hat{\mu }$ and $\hat{\sigma }$ are estimates of the common baseline and standard deviation for all pixels in an image time series. We use the median of the pixel-specific estimates in order to be robust against extreme values,

$$\hat{\mu }=Median({\hat{\mu }}_{i})\;and\;\hat{\sigma }=Median({\hat{\sigma }}_{i}).$$

We observed that for low SNRs there was a positive correlation between SNR and the number of events detected with the ROA algorithm. If a typical dataset was smoothed to attain at least an SNR of 9, this correlation disappeared. In order to attain this desired level of SNR of at least 9, we perform a parameter search of varying levels of spatiotemporal smoothing and apply spatial Gaussian smoothing and temporal boxcar smoothing. Although we cannot guarantee that an SNR of at least 9 is the optimal target for datasets acquired by different labs and hardware, an SNR of at least 9 worked well for the external datasets we tested. For the time series *X_i_*(*t*), the temporal smoothing is applied by binning frames and not as a moving average. The parameter search may be performed on all imaging trials independently or on a selected trial serving as a template for the rest of the analyses. For spatial smoothing, we perform a parameter search where the sigma of the Gaussian filter is increased from 0 to 2 pixels. If the desired SNR is not reached by spatial filtering alone, we keep the maximum spatial smoothing and do a parameter search for the number of averaged frames between one and 30 frames. If the target SNR is still not reached, the configurations are set to 2 pixels for the spatial smoothing and 30 frames for the temporal smoothing. We perform the parameters search for the number of frames to average by using the interval halving method. To limit the computation time in large time series data only the last 1,000 merged frames are used to compute the SNR in the parameter search. The spatial and temporal smoothing parameters can also be set manually. The parameters are estimated and stored for interactive threshold adjustment and filtering when the pre-process button in the GUI is pressed.

### Optional Threshold Adjustment and Filtering Results

As the size and duration of two-photon microscopy of astrocytic Ca^2+^ events follow a power law distribution and the optical resolution of two-photon microscopy is an order of magnitude poorer than the smallest astrocytic processes, there is a continuum between signal and noise. Moreover, we do not have access to the ground truth of real-life data. Consequently, the threshold applied will be somewhat heuristic (we have chosen four times the standard deviation of the noise as default: *κ* = 4) and the events detected will be strongly dependent on the threshold applied. In addition, no matter the threshold applied, a large proportion of the true Ca^2+^ event will go undetected due to the limitations of (non-super-resolution) optical microscopy. For these reasons it is desirable to be somewhat conservative when deciding what is signal and what should be considered noise, and a tool is provided for interactively changing the threshold by adjusting *κ* and filtering out ROAs below a minimum size and duration.

### Performance

The main outputs from Begonia can be found in Table 1. The ROA method and surrounding pipeline have been created to support the analysis of large datasets on moderate performance computers like a personal laptop. Two-photon recordings at high frame rates typically store data in integer formats at rates of ~1 GB per minute. However, for many calculations, data need to be transformed to floating-point formats. These can be two to eight times more memory intensive and consequently exhaust the working memory of the computer even for shorter (5–10 min) recordings. For these reasons, in the pipeline where the sheer size of the file may exceed the capacity of a normally configured computer, data are chunked and analyzed in smaller segments. Moreover, data retrieval is implemented with lazy (on-demand) reading. On a personal laptop, with 16 GB RAM, it took 15, 31, 51, 143, and 267 s, to analyze 100, 500, 1,000, 2,500, and 5,000 frames of 512 × 512 pixels, respectively.

**Table 1:** The primary outputs from Begonia and where to find them.

Output	Description
ROA event table	Each row represents one event. The table contains the center position, start frame, end frame, duration, size and duration. Saved with the key-value pair “roa_table” after processing.
ROA traces	Time series of roa frequency (new events per frame) and ROA density (active x−y−t voxels) key-value pair “roa_traces” after processing.
ROA mask	A binary 3D matrix representing the imaging time series, where 1’s represent detected events. Saved with the key-value pair “roa_mask_chx” (*x* denoting the channel where ROAs have been detected).
ROA 3D plot	A GUI is provided to produce 3D ROA plots as in Figure 5B.
ROI table	A table containing the manually segmented ROIs. Each row represents one ROI and contains the size, location, channel, name and a unique identifier of the ROI. Saved with the key-value pair “roi_table.”
ROI traces	Raw and Δ*F/F*_0_ normalized signals from ROIs. Saved with the key-value pair “roi_signals_raw” and “roi_signals_dff.”
ROI active pixels	Fraction of ROI that has a ROA per time unit. Saved with the key-value pair “roi_signals_raw” and “roi_signal_rpa.”

## Results

### Workflow

We built Begonia with the flow of data in mind, and a high priority during development was to create responsive GUIs and speed up functions that have high computational demands. Furthermore, time-consuming steps can be queued and processed in batch operations. The workflow is outlined in Figure 1. The intended workflow is a step-by-step procedure where small chunks of the data are processed at a time and the results and intermediate data are stored with data locations associated with the imaging time series object. The chunking ensures that the software can be applied to (infinitely) large datasets whereas saving intermediate data adds flexibility. For example, if there is a failure during one or more processing steps, the fault can be troubleshot and the procedure can be started from the point where it stopped. Also, intermediate steps can easily be reprocessed using different parameters.

The first step of the workflow in Begonia is to identify imaging time series data and instantiate classes to interact with them. Begonia includes methods that search directories for supported two-photon imaging formats and returns a list of imaging objects. The objects are used to access the imaging time series data and microscope metadata in a standardized way, even if the recordings are stored using heterogeneous file formats. Instructions on how to make new imaging classes for unsupported formats are provided in the documentation.

Begonia provides a general method to save data together with the imaging data in folders in the directory tree. The data in these folders can then be directly accessed via the imaging objects and are known in this toolbox as data locations. Data stored through the data locations system may be retrieved via the imaging time series objects using keywords.

**Figure 3:**
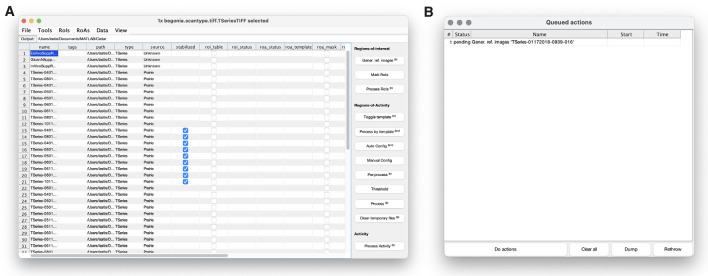
Begonia includes a data management GUI with **(A)** a main window where rows represent data entities (typically imaging data) and columns contain corresponding metadata. Actions in the main window can be run immediately or be added as tasks to a processing queue **(B)** that can be loaded for time-consuming batch processing.

We have provided a workflow GUI (Figures 1B, 3A) that lists groups of data locations and associated metadata in a table and allows the user to select items and run procedures. It includes basic steps typically performed in the analysis of two-photon imaging data, and can easily be expanded or modified to allow for other types of analyses on the data. The user can select and pass single or multiple data objects to functions, other GUIs or to the batch processing queue (Figure 3B). Standard procedures that are currently implemented in the toolbox are image alignment using the NoRMCorre software package (Pnevmatikakis and Giovannucci, 2017), manual segmentation of ROIs in a GUI, and running the ROA algorithm.

The last step of the processing workflow is to combine imaging data with other types of data (e.g., behavioral data) and to export data. Begonia provides a way to easily combine the results from the analysis of the imaging data with other types of data (for instance electrophysiology or behavioral metrics) in a data class called MultiTable. The MultiTable enables you to resample, align and slice your dataset to export a desired subset of data for plotting or statistical analyses (Figure 1D and see “Materials and Methods” section). The main outputs from Begonia is listed in Table 1.

### Data Management and Processing

Begonia is built around the concept of data locations to save metadata and derived data throughout the analyses (see “Materials and Methods” section). Begonia further provides a template GUI for working with these abstract representations of the data and metadata (Figure 3). Here, DataLocation objects, e.g., a two-photon microscopy time series with corresponding metadata, appear as an entity in a list. The GUI enables the user to selectively see metadata coupled to the data location objects in the same list, and gives quick access to pass these objects to the MATLAB workspace or functions represented by buttons and menu options. You may add new buttons and menus with associated functions to the GUI by passing anonymous functions as input arguments during the initialization of the GUI. The GUI also provides a processing queue (Figure 3B), where actions on the data location objects can be visualized before being executed in a batch-wise process.

### Marking ROIs

**Figure 4:**
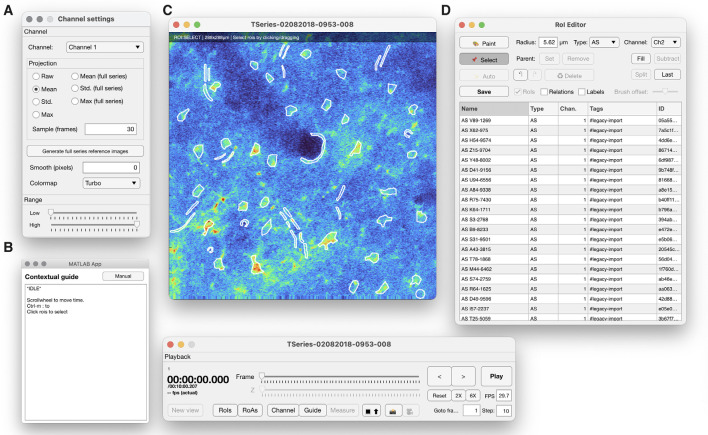
The ROI manager provided in Begonia comprises the following features: **(A)** a view controller that determines what and how imaging data should be displayed, **(B)** a contextual guide window that informs the user of useful shortcuts and keyboard commands for the current mode. **(C)** The main image window, where raw imaging data or running averages or various projections of the imaging data can be displayed. Manually segmented ROIs are drawn into the overlay. **(D)** An ROI editor that organizes the ROIs and enables the user to classify, rename, delete or modify ROIs.

The toolbox provides a multi-purpose GUI for visualization of imaging data, ROAs, and manual segmentation of ROIs (Figure 4). The GUI components may also be assembled for other types of analyses. Imaging data may be visualized as raw data, running average data, or projections of the whole time series. ROIs are manually drawn with a paintbrush tool and saved in a list where the type of ROI and relationships between ROIs may be defined.

### Region of Activity (ROA) Analysis

The ROA algorithm is an event-based method to quantify astrocytic Ca^2+^ signals. An earlier iteration of the ROA algorithm was applied to two-photon microscopy data in Bojarskaite et al. (2020). In the current version, the noise estimation strategy has been adapted from Wang et al. (2019). The analysis comprises first a spatial and temporal filtering of two-dimensional time series data to attain an acceptable SNR, followed by a pixel-by-pixel quantification of noise over time, to define a threshold for signal detection. The steps of the method are shown in Figure 2. The appropriate level of smoothing is automatically found as the first step of the algorithm (see “Materials and Methods” section). This part of the algorithm enables the ROA method to be run with few input parameters and still give reproducible results.

**Figure 5:**
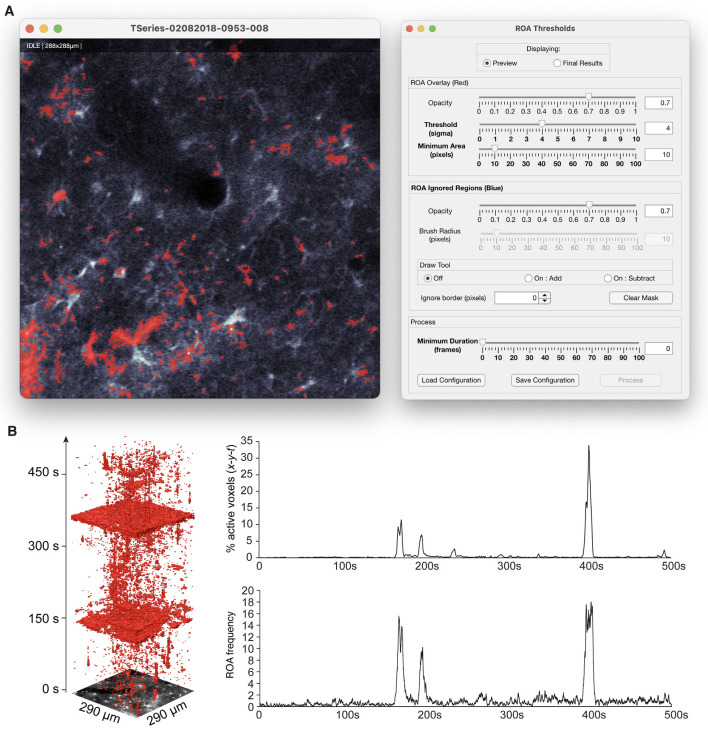
ROA thresholding and output. **(A)** The thresholding step of the ROA algorithm is supported by a GUI that displays an overlay of the ROAs on top of the imaging data, and allows for interactive adjustment of the Ca^2+^ event detection threshold, adjusting the minimum size and duration of events, and defining regions that should be ignored. **(B)** An *x-y-t* volume rendering of a time series of astrocytic Ca^2+^ signals and corresponding traces of ROA density (% active voxels) and ROA frequency events per minute per μm^2^.

The ROA analysis can be executed by running a few functions in series and the results can be viewed in a similar fashion. The ROA analysis is simplified by using the data management GUI which guides the analyst through the following steps: (1) loading imaging data into the GUI by searching specified directories for supported imaging formats; (2) setting the two smoothing parameters by either clicking “Auto config” or “Manual config”; (3) running the pre-processing by clicking the “Pre-process” button; (4) previewing detected events and adjusting the detection threshold in the ROA GUI (Figure 5) by clicking the “Threshold” button. The GUI also enables filtering small and short events as well as ignoring regions around the edge of the FOV or other user-defined areas; and (5) converting the detected, filtered events to time series traces of (a) the density of Ca^2+^ signal events per time unit and (b) the number of new events per frame. Metrics about the events, such as size, duration, and timing, are saved in a table where each row represents one event.

This list of output of the ROA algorithm is less extensive than that of the AQuA algorithm (Wang et al., 2019). Most importantly, AQuA provides information about the spatial dynamics of every single Ca^2+^ event and applies a set of rules to separate Ca^2+^ events that may be splitting into several events, or merging into larger events. The overall performance of the ROA algorithm was compared to the AQuA algorithm using a downsampled dataset detecting the same trends in Ca^2+^ signaling across different sleep stages in Bojarskaite et al. (2020).

### ROA Activity in ROIs Analysis

As there is substantial evidence that the astroglial subcompartments behave differently, separate analyses of anatomical subcompartments are warranted (Bazargani and Attwell, 2016). The ROA analysis output does not disclose the underlying anatomical structure of the tissue. Therefore the output of the ROA algorithm can be filtered based on manually defined ROIs providing the percentage of an ROI or subcompartment active at a given time.

### Accessibility

The Begonia toolbox may be downloaded at https://github.com/GliaLab/Begonia. A detailed user manual and links to third party software packages and links to instructional videos are provided there as well as a user community discussion group.

## Discussion

Deciphering astroglial Ca^2+^ signals remains one of the biggest challenges for the field of glioscience (Semyanov et al., 2020). Even though a plethora of functions are thought to be supported by astroglial Ca^2+^ signals, the interpretation and importance of these signals are still somewhat controversial (Bazargani and Attwell, 2016; Shigetomi et al., 2016). When studying unanesthetized awake-behaving mice, a range of Ca^2+^ signals can be detected, from small events close to the level of noise to global increases in astrocytic Ca^2+^ signaling across the cortex in relation to neuromodulatory activity (Srinivasan et al., 2015; Bojarskaite et al., 2020). To better characterize this wide range of event types is a key first step in identifying their role in the circuitry, and may contribute to solving some of the controversies in this field. Here, we present a toolbox tailor-made for the analyses of two-photon microscopy data of astrocytic Ca^2+^ signals in conjunction with rich behavioral data, from raw data to aggregated results in tables.

A large proportion of astroglial Ca^2+^ signals are likely stochastic events (Semyanov et al., 2020), and under certain conditions, they are quite infrequent (Bojarskaite et al., 2020). In these cases, long acquisition times are warranted (e.g., time series of 10 min or more) to accurately quantify event rates and dynamics. Furthermore, when imaging awake mice, a high frame rate is warranted to be able to effectively remove movement artifacts (Pnevmatikakis and Giovannucci, 2017). For these reasons, astroglial Ca^2+^ imaging time series from awake mice are often large files up to tens of gigabytes. We have therefore gone to great lengths to optimize Begonia’s processing to run quickly on large files even on moderate performance computers. The workflow has been organized in such a way that the time-consuming steps of the analyses can be performed unsupervised. Moreover, an important goal with the toolbox is responsive behavior and a short waiting time when manually interacting with the data. Therefore all large data in the pipeline are loaded lazily, i.e., on request. Even so, some of the analyses provided in Begonia will be slow to execute with large recordings due to the sheer number of calculations performed. Incorporating hardware-accelerated analyses could hold great potential for some of these time-consuming steps in the future.

The first hurdle in the analyses of two-photon microscopy data is to import the imaging data in an efficient fashion to the analysis platform. Two-photon microscopy data are typically stored as TIFF files (either single-frame files or multi-page TIFFs). Even so the TIFF format allows for many variations and data from different channels, trials and most importantly metadata are saved in different ways by different setups. Consequently, there are no standardized ways to read two-photon microscopy data across platforms. ImageJ and FIJI offer a low threshold plug-and-play software that can handle many different TIFF implementations but has the drawback of confining the analyses to the ImageJ framework (Schindelin et al., 2012; Schneider et al., 2012). Begonia offers direct support for imaging data from ScanImage and PrarieView software as well as TIFF files read by the TIFFStack (Muir and Kampa, 2014) library, but just as important provides an API for easy adaptation of other imaging data formats to our pipeline.

**Figure 6:**
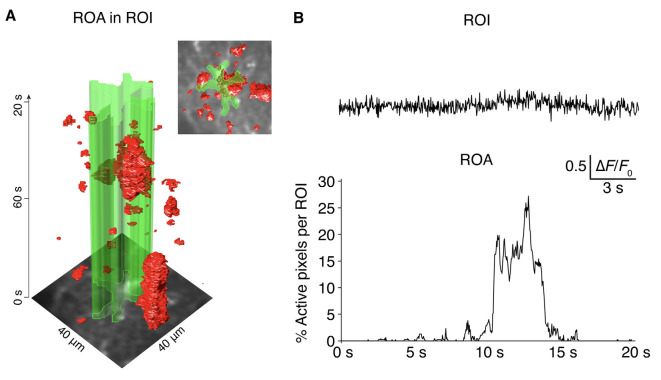
ROA algorithm vs. ROI analyses. **(A)** The complex spatiotemporal distribution of astroglial Ca^2+^ signals presented as an *x-y-t* volume rendering. The outline of an astrocyte (ROI) is presented in green. **(B)** The ROA algorithm is considerably more sensitive than a standard ROI-based analysis, as evident when comparing the percentage of active pixels detected with the ROA algorithm within the ROI defined in **(A)**, compared to the extracted mean fluorescence from the ROI defined in **(A)** where no signals would be detected with a standard peak detection algorithm.

In this article, we present an event-based Ca^2+^ signal detection tool for the unbiased quantification of astrocytic Ca^2+^ signals with a high level of detail. The algorithm separates Ca^2+^ signals from the noise for each individual pixel over time, before connecting the active *x-y-t* voxels to Ca^2+^ signal events. The ROA algorithm performs considerably better than static ROI analyses in terms of sensitivity and accuracy (see Figure 6 and Bojarskaite et al., 2020). An earlier iteration of this algorithm was used for the analyses of Ca^2+^ signaling data in Bojarskaite et al. (2020). The present algorithm calculates the threshold for signal detection slightly differently, similar to that of the AQuA algorithm (Wang et al., 2019). The event definition of the ROA algorithm and the first part of the AQuA algorithm share many similarities, but there are also noteworthy differences. In our hands, the AQuA method had some limitations that made it impractical or even impossible to use for our large files of high frame rate imaging data (Bojarskaite et al., 2020). The first issue was processing time. For our long (e.g., 18,000 frames), 30 Hz two-photon microscopy data, processing with the AQuA algorithm failed even on a high-performance computer (128 Gb RAM, 18 cores) due to running out of RAM. When run on a moderate performance computer, a moderately sized dataset of 5,000 frames recording that ran in 267 s with the ROA algorithm took ~2 full days to analyze with the ROA algorithm. Moreover, the AQuA algorithm did not perform well in terms of separation of signal from the noise in our data, that had a poor SNR due to our high acquisition rates, resulting in high levels of artifactual events detected. The ROA algorithm is considerably less extensive than the AQuA algorithm as it does not analyze the spatiotemporal dynamics of each signaling event separately. Rather it only reports on the frequency of starting events and density of events per time unit in the full FOV or per manually segmented compartments, as well as descriptive measures of maximum spatial extent and duration of individual ROAs. This in part explains the large difference in processing times between the two algorithms—i.e., the ROA algorithm is a substantially more lightweight algorithm.

The size and duration of astrocytic Ca^2+^ events are known to follow a power law distribution (Jung et al., 1998; Wu et al., 2014; Semyanov et al., 2020), and Ca^2+^ signals in the smallest processes and leaflets are of considerably smaller spatial extent than the resolution limit of optical (non-super resolution) microscopy allows. Therefore, even minute changes in the settings and consequently the thresholds and filtering applied will profoundly change the distribution of Ca^2+^ signal events detected. These reasons call for being conservative when determining the threshold for separating signal and noise and is also an argument for not considering the smallest detected events in a given recording. An interactive tool is provided that enables easy adjustment of the threshold applied and the minimum sizes and durations allowed for a Ca^2+^ event.

One goal with the ROA method was to provide an algorithm that could be used without too many input parameters to enable a more direct comparison between datasets recorded with different hardware or settings. We have therefore provided a pre-processing method that evaluates the imaging data and determines what filtering is appropriate to: (a) ensure an accurate identification of events with few artifacts; and (b) to enable more direct comparisons between data from different datasets. The pre-processing will find suitable parameters for spatial and temporal smoothing until an empirically chosen level of SNR of at least nine is attained. We have tested the algorithm on datasets acquired in three different laboratories with different types of hardware and acquisition parameters. Even so we cannot be certain that the target SNR of nine is optimal for two-photon image recordings of all types, as photon statistics and data quality vary significantly across acquisition hardware, fluorophores, and experimental protocols.

In principle, other types of fluorescent data than astrocytic Ca^2+^ signals could be possible to analyze with Begonia. The pipeline already supports signal extraction and neuropil subtraction from neuronal ROIs (accessible through the API). Moreover, the ROA algorithm could prove useful in the future for quantifying both neuronal Ca^2+^ signals or other types of dynamic fluorescent sensors but has not been validated for this purpose yet.

To the best of our knowledge, no algorithm exists for automatically segmenting images of astroglial cells into their respective subcellular compartments of somata, processes, and endfeet. This is also true for our ROA method. Therefore, we provide the option of integrating manually segmented ROIs with the automatic ROA algorithm, such that subcompartment specific ROA signals can be described separately. Ideally ROIs should have been detected and correctly classified without manual intervention. Potentially, machine learning algorithms, using manually segmented ROIs as a training dataset could provide such functionality in the future.

Our toolbox requires imaging data to be adapted to standardized classes and be funneled through our data management pipeline. We believe this is a major strength for this toolbox as it allows for more efficient and flexible approaches to data management. This may, however, also be perceived as a potential drawback—the tools in our toolbox are not standalone pieces of software that take general image formats and directly outputs results—rather, the tools are embedded in a workflow pipeline that must be executed in a certain fashion. Consequently, the threshold to start using the toolbox could be somewhat higher. To mitigate these problems, we have made instructional videos and live script example cases to be run with example datasets to quickly familiarize potential users with the API. GUIs are also provided for users that are not comfortable scripting their analyses. The Begonia software published here is the first version of this package, built to be easily extendable, and we hope that the project will evolve both locally and in collaboration with potential external users through the GitHub repository and associated user community discussion group.

## Data Availability Statement

Publicly available datasets were analyzed in this study. Links to the example datasets can be found here: http://github.com/GliaLab/Begonia.

## Ethics Statement

The animal study was reviewed and approved by the Norwegian Food Safety Authority.

## Author Contributions

KV and RE: conceptualization and funding acquisition. DB, KÅ, EH, KP, CC, GH, and RE: methodology. RE: resources. DB, KÅ, LB, CC, GH, and RE: writing—original draft. LB and EH: acquiring microscopy data and testing. DB, KÅ, EH, KP, KV, LB, CC, GH, and RE: writing—review and editing. KP, KV, and RE: supervision. All authors contributed to the article and approved the submitted version.

## Conflict of Interest

The authors declare that the research was conducted in the absence of any commercial or financial relationships that could be construed as a potential conflict of interest.
